# The effect of *Tmem135* overexpression on the mouse heart

**DOI:** 10.1371/journal.pone.0201986

**Published:** 2018-08-13

**Authors:** Sarah Aileen Lewis, Tetsuya Takimoto, Shima Mehrvar, Hitoshi Higuchi, Anna-Lisa Doebley, Giangela Stokes, Nader Sheibani, Sakae Ikeda, Mahsa Ranji, Akihiro Ikeda

**Affiliations:** 1 Department of Medical Genetics, University of Wisconsin-Madison, Madison, Wisconsin, United States of America; 2 Institute for Innovation, Ajinomoto Co., Inc., Tokyo, Japan; 3 Department of Electrical Engineering, Biophotonics Laboratory, University of Wisconsin, Milwaukee, Wisconsin, United States of America; 4 Department Ophthalmology and Visual Sciences, Biomedical Engineering, and Cell and Regenerative Biology, University of Wisconsin-Madison, Madison, Wisconsin, United States of America; 5 McPherson Eye Research Institute, University of Wisconsin-Madison, Madison, Wisconsin, United States of America; University of Cincinnati College of Medicine, UNITED STATES

## Abstract

Tissues with high-energy demand including the heart are rich in the energy-producing organelles, mitochondria, and sensitive to mitochondrial dysfunction. While alterations in mitochondrial function are increasingly recognized in cardiovascular diseases, the molecular mechanisms through which changes in mitochondria lead to heart abnormalities have not been fully elucidated. Here, we report that transgenic mice overexpressing a novel regulator of mitochondrial dynamics, transmembrane protein 135 (*Tmem135*), exhibit increased fragmentation of mitochondria and disease phenotypes in the heart including collagen accumulation and hypertrophy. The gene expression analysis showed that genes associated with ER stress and unfolded protein response, and especially the pathway involving activating transcription factor 4, are upregulated in the heart of *Tmem135* transgenic mice. It also showed that gene expression changes in the heart of *Tmem135* transgenic mice significantly overlap with those of aged mice in addition to the similarity in cardiac phenotypes, suggesting that changes in mitochondrial dynamics may be involved in the development of heart abnormalities associated with aging. Our study revealed the pathological consequence of overexpression of *Tmem135*, and suggested downstream molecular changes that may underlie those disease pathologies.

## Introduction

Tissues with high-energy demand, such as the retina, brain, skeletal muscle, and heart, are rich in the energy-producing organelles, mitochondria, and sensitive to mitochondrial dysfunction [1]. In the heart muscle, 35% of the volume of each cardiomyocyte is composed of mitochondria [2]. Thus, global disruptions to mitochondrial function have detrimental effects on the function of the heart and lead to a number of heart disorders including cardiomyopathies, arrhythmias and heart failure [3,4]. Decline in the mitochondrial function is also observed in the aging heart [5,6], which is thought to reduce the ability of cardiomyocytes to counter pathological stress. It has been shown that mitochondria are dynamic organelles undergoing fission and fusion (mitochondrial dynamics). Balanced mitochondrial dynamics are particularly important in terms of the quality control of this organelle, where fusion enables compensation for mitochondrial damage by joining neighboring mitochondria, and fission allows for elimination of mitochondria with unrecoverable damage through autophagy (mitophagy) [7]. Accordingly, balanced mitochondrial dynamics are essential for tissue homeostasis. Indeed, dysregulation of mitochondrial dynamics in the heart has been shown to result in abnormalities including cardiomyopathy and heart failure [8,9]. Ablation of a mitochondrial fission factor, dynamin-related protein 1 (*Drp1*) in the adult mouse heart lead to mitochondrial enlargement and dilated cardiomyopathy [10]. On the other hand, impairment of mitochondrial fusion through combined ablation of mitochondrial fusion factors, mitofusin (*Mfn*) 1 and *Mfn2*, in the adult mouse heart caused mitochondrial fragmentation and eccentric cardiac hypertrophy [10]. These observations indicate that proper regulation of mitochondrial dynamics is essential for heart health. However, the molecular mechanisms through which changes in mitochondrial dynamics lead to heart abnormalities have not been fully explored.

Recently, we discovered a novel regulator of mitochondrial dynamics, transmembrane protein 135 (TMEM135) [11] which is highly conserved across species [12]. TMEM135 regulates mitochondrial morphology and dynamics, presumably through promoting mitochondrial fission [11]. A mouse mutation in *Tmem135*, a point mutation which results in an early stop codon, causes elongated mitochondria with defective respiratory functions, which leads to age-dependent retinal abnormalities with early onset and faster progression [11]. On the other hand, overexpression of *Tmem135* transgene under the control of chicken beta-actin promoter (*Tmem135* TG) leads to fragmented mitochondria with reduced basic oxygen consumption rate and ATP production in fibroblasts [11]. Here, we report that *Tmem135* TG mice show disease phenotypes in the heart including hypertrophy and collagen accumulation. To identify key molecules and pathways affected in *Tmem135* TG hearts, we examined gene expression changes in the *Tmem135* TG heart combined with gene ontology analysis. Our study identifies a pathway through which abnormal mitochondrial dynamics, particularly excessive mitochondrial fission, may lead to disease phenotypes in the heart.

## Materials and methods

### Mice

All experiments were performed in accordance with the National Institute of Health Guide for the Care and Use of Laboratory Animals and were approved by the Animal Care and Use Committee at the University of Wisconsin-Madison. *Tmem135* TG mice were generated at University of Wisconsin-Madison as previously described [11]. We replaced the EGFP sequence in the pCX-EGFP vector (kindly provided by Dr. Junichi Miyazaki [13]) with the full length *Tmem135* cDNA and named it *pCX-Tmem135*. We used *pCX-TMEM135* for the transgene construct after linearization with HindIII and SalI (New England Biolabs, Ipswich, MA). The construct was micro-injected into pronuclei of FVB/NJ embryos at the Transgenic Facility of the University of Wisconsin-Madison Biotechnology Center. Transgene-positive founders were crossed to C57BL/6J mice for one generation and subsequently maintained by intercrossing (FVB/NJ—C57BL/6J mixed genetic background). For the purpose of examining retinal phenotypes in separate studies, we removed the *Ped6b*^*rd1*^ mutation in the FVB/NJ background known to cause retinal degeneration during this process [11]. The *Tmem135* transgene maps to chromosome 19 between SNPs 19-025120427-M and 19-049914266-M. For experiments, *Tmem135* TG mice as well as age-matched, littermate non-transgenic control (wild-type [WT]) mice were used.

### Heart weight/ body weight ratio

Body weights were measured in grams using an Ohaus CS 200 scale (Ohaus corporation, NJ). Heart weights were measured in mg immediately following dissection using a Pinnacle Balances P-114 scale (Denver Instruments, Bohemia, NY). Ratios were calculated by dividing the heart weight by the body weight (n = 6 WT, n = 9 TG).

### Masson’s Trichrome Staining

Following asphyxiation by CO_2_ administration, hearts were immediately removed and immersion fixed in Bouin’s fixative overnight at 4°C. Hearts were then rinsed and dehydrated, and embedded in paraffin. Paraffin blocks were sectioned 6 μm thick on an RM 2135 microtome (Leica Microsystems, Wetzlar, Germany) and mounted on glass slides. Three non-consecutive, central sections of each heart were chosen for staining. Central sections contained all 4 chambers. The slides were stained with Masson’s Trichrome Stain Kit (American MasterTech, Lodi, CA) to distinguish heart muscle cells, nuclei and collagen according to the manufacturer’s protocol. Masson’s Trichrome-stained sections were imaged on an Axio Imager 2 Microscope and camera (Carl Zeiss MicroImaging, Thornwood, NY). Collagen positive areas were quantified using the threshold function of ImageJ 1.46r [14], and represented as % blue area of total area measured, which were averaged for each genotype (n = 4 WT, n = 4 TG).

### Immunohistochemistry

For immunohistochemistry on cryostat sections, 6-μm-thick sections were blocked in PBS with 0.5% Triton X-100 and 2% normal donkey serum for 20 min at room temperature. Next, sections were incubated at 4°C overnight in primary antibody solution. Primary antibodies against Collagen III (Abcam, Cambridge, MA) and myosin (Sigma, St. Louis, MO) were used. Sections were rinsed in PBS and incubated with a secondary antibody in block solution (1:200 dilution) for 2 hours at room temperature. Following PBS rinse, sections were stained with 4’,6-diamidino-2-phenylindole (DAPI). All sections were imaged on the Nikon A1R+ confocal microscope (Nikon, Tokyo, Japan) equipped with high sensitivity GaAsP detectors; high-speed resonant scanner; six lasers at 405, 440, 488, 514, 561, and 640 nm. NIS-Elements AR software (Nikon) was used for image acquisition and image analysis.

### Quantification of cardiomyocyte size

Following asphyxiation by CO_2_ administration, hearts were immediately dissected and rinsed with PBS. Hearts were then fixed in 4% paraformaldehyde by cannulation and immersion for two hours and dehydrated before embedding in paraffin. Paraffin blocks were sectioned 6 μm thick on an RM 2135 microtome (Leica Microsystems, Wetzlar, Germany) and mounted on glass slides. Three nonconsecutive, central sections of each heart were chosen for staining. Sections were rehydrated to PBS and heated in citric acid buffer for antigen retrieval. Sections were then stained with FITC-labeled wheat germ agglutinin (WGA) (Sigma, L4895) in PBS (1:200 dilution) to distinguish the cell membrane of cardiomyocytes. The size of cardiomyocytes was measured as areas outlined by WGA staining. The surface area of at least 250 transverse sections of cardiomyocytes were measured per heart section and averaged for each animal (n = 5 WT, n = 6 TG).

### Quantification of mitochondrial size

Following asphyxiation by CO_2_ administration, hearts were immediately dissected into fixative (2% glutaraldehyde, 4% paraformaldehyde, 1% osmium tetroxide). The left ventricular tissue was cut into sections no larger than 1mm thick and fixed overnight at 4 degrees. The tissue was post-fixed in 1% osmium tetroxide and dehydrated to 100% ethanol. The tissue was then washed in propylene oxide before being embedded in 100% propylene oxide and baked to cure resin. Sections were then cut to 0.1 μm and stained with uranyl acetate. Sections were imaged using Philips CM120 STEM. Cardiomyocytes containing correct orientation of z lines were imaged for mitochondria. Surface area of mitochondria were measured using ImageJ software (n = 4 WT, n = 4 TG; 8655 WT mitochondria and 8950 TG mitochondria examined).

### Gene expression analysis

We conducted RNA sequencing analysis to compare gene expression profiles between 6-month-old *Tmem135* TG and WT mouse hearts (n = 3 WT, n = 3 TG). The left ventricular tissue was dissected and snap frozen in liquid nitrogen. The heart tissue was first homogenized using the Qiagen Tissuelyser II system with stainless steel beads (Qiagen USA, Germantown, MD). RNA was extracted in TRIzol (Thermo Fisher Scientific, Waltham, MA) and chloroform (Thermo Fisher Scientific) was used to separate the aqueous layer. Samples were purified using the Qiagen RNeasy Mini Kit protocol according to manufacturer’s instructions (Qiagen). Stranded mRNA was sequenced using the sequencing platform HiSeq2000 (Illumina, San Diego, CA) at University of Wisconsin-Madison Biotechnology Center. Raw, 100 bp reads were examined for quality using FastQC v0.10.1 [15]. Reads were trimmed with fastx-toolkit v 0.0.14, aligned with tophat v2.0.9 [16,17] and converted to bam files using samtools v0.1.19 [18]. Using the Cuffdiff [17] software (v2.1.1), we detected differentially expressed (DE) genes (q-value < 0.05) in the *Tmem135* TG heart compared to WT controls. Gene expression data are available at the Gene Expression Omnibus (GEO), accession number GSE99522. Using the Database for Annotation, Visualization, and Integrated Discovery Functional Annotation Tool (DAVID) for gene ontology (GO) term analysis [19,20], we categorized differentially expressed (DE) genes in the *Tmem135* TG heart. We obtained gene expression data of the heart of aged (25–28 month old) C57BL/6J mice (GSE12480), and a diet-induced obese C57BL/6J mice (17 week old) (GSE47022) [21] from a public database [22]. Then, we determined DE genes in the heart of each mouse model compared with control mice (young [4–6 month old] C57BL/6J mice and non-obese age-matched C57BL/6J mice on control diet, respectively). Overlap between DE gene sets was determined using *GeneOverlap* simulation in R and significance was determined using fisher’s exact test.

### Western blot analysis

Following asphyxiation by CO_2_, the heart was collected and stored at -80°C. The heart was homogenized with RIPA buffer (Thermo Fisher) containing protease/phosphatase inhibitor cocktail (Thermo Fisher) on ice. Proteins were separated by SDS-PAGE gel electrophoresis and transferred to a PVDF or PVDF-FL membrane. To detect each protein, the membrane was incubated with a primary antibody for ATF4 (Cell Signaling Technology [CST]), ATF6 (Novus Biologicals, Littleton, CO), IRE1alpha (CST), PERK (CST), Phospho-PERK (Thr981) (Elabscience Biotechnology), GRP78/Bip (CST), Pdi (CST), CHOP (CST), phospho-eIF2alpha (CST), eIF2alpha (CST), beta-actin (Hybridoma Bank, Iowa City, IA), and then with a horseradish peroxidase (HRP)-conjugated secondary antibody (1:10000 dilution) or LI-COR secondary antibody (LI-COR, Lincoln, NE) for florescent detection (1:10000). LI-COR secondary antibody was detected using the LI-COR Odyssey System. HRP-conjugated secondary antibodies were detected using enhanced chemiluminescent substrates for HRP (ThermoFisher Scientific, USA), followed by exposure of the X-ray film. Films were imaged with Gel Doc XR System (Bio-Rad Laboratories Inc., Hercules, CA). The optical density of each band was measured by ImageJ.

### Optical cryo-imaging

Hearts were collected for the 3D cryo-imaging protocol (n = 7 WT, n = 7 TG). The snap-frozen hearts, stored at −80°C until the day of study, were imaged in our custom-made cryo-imager at the Biophotonics Lab, University of Wisconsin-Milwaukee. We have reported extensively on the cryo-imaging system in previous studies [23–25]. In brief, a microtome blade sequentially slices the embedded tissue. For each slice, the autofluorescence images from reduced nicotinamide adenine dinucleotide (NADH) and flavin adenine dinucleotide (FAD) were captured to measure the redox state of the mitochondrial metabolism in the tissue. Major NADH and FAD signals captured originate from mitochondria with negligible contributions from cytoplasmic sources [26,27]. The 3D rendered images using z-stacks of all the image slices for both NADH and FAD signals, and the NADH to FAD redox ratio of each heart was calculated voxel by voxel as previously explained [23–25]. The intensity histogram distribution of redox ratio (NADH/FAD) for each heart was determined, and the mean value (RR) calculated as a quantitative marker for the oxidative state of the tissue.

### Amino acids analysis

The heart tissue (20 mg) was mixed with 150μL of 10% (w/w) trichloroacetic acid, and centrifuged immediately (4°C, 30 min, 10000 g) to remove precipitated protein. All samples were kept on ice to minimize chemical reactions of thiol metabolites. The amino acid concentrations were measured by an automatic amino acid analyzer (L-8800; Hitachi, Tokyo, Japan). Briefly, amino acids, separated by cation exchange chromatography, were detected spectrophotometrically after post-column reaction with the ninhydrin reagent.

## Statistical analysis

Statistical analyses were performed using GraphPad Prism software (GraphPad Software, Inc., San Diego, CA). Significance of the difference between two groups was calculated by unpaired Student’s two-tailed t-test. Variance in the heart weight to body weight ratio was compared by F-test. Comparison of survival curves was performed by Mantel-Cox log-rank test as well as Gehan-Breslow-Wilcoxon test. The size distribution of mitochondria was compared by the Kolmogorov-Smirnov test.

## Results

**Fig 1:**
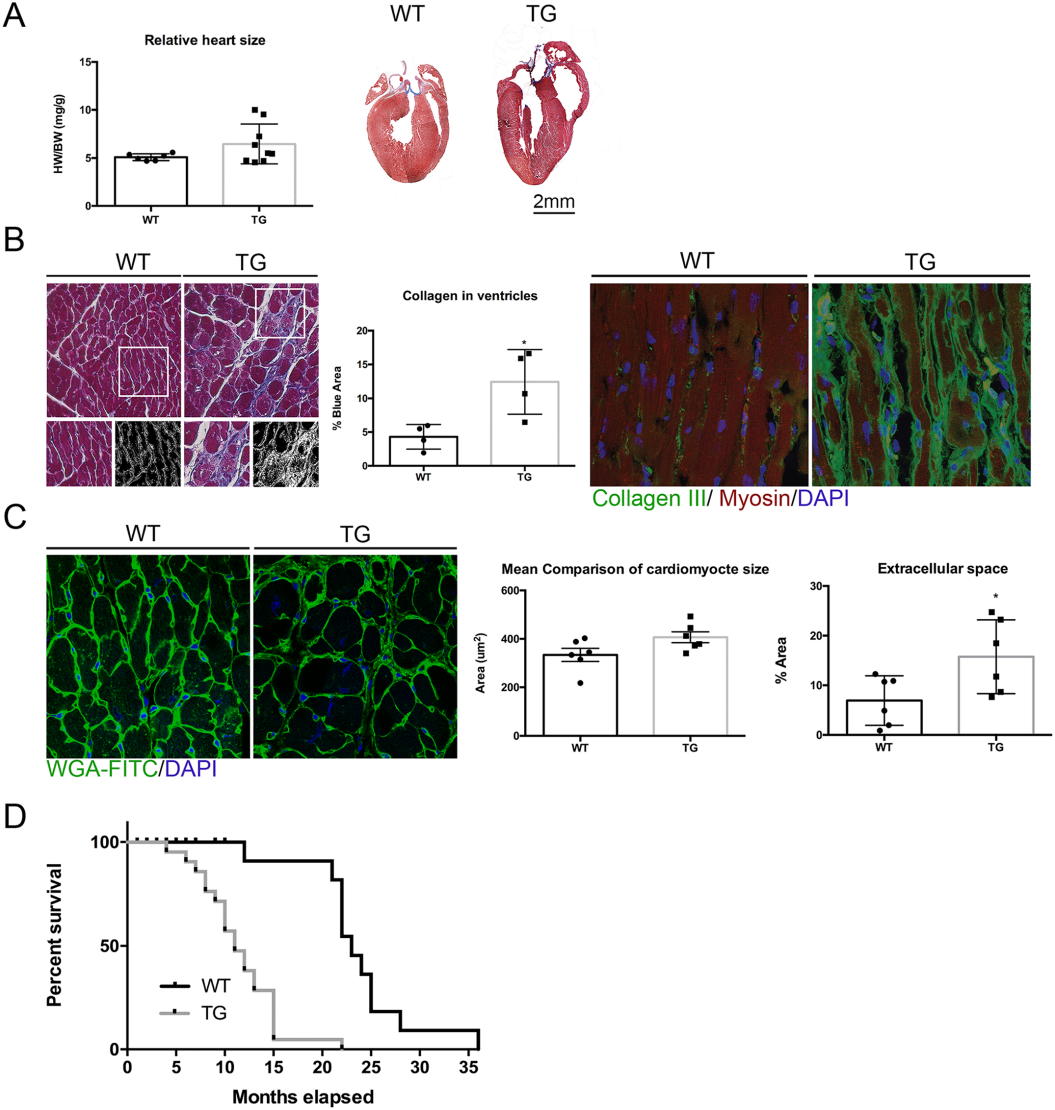
*Tmem135* TG mice display disease phenotypes in the heart. A) Heart to body weight ratios of 14 month old mice show a trend of increase in *Tmem135* TG mice indicative of mild cardiac hypertrophy (n = 7 WT, n = 9 TG; p = 0.085 by t-test). Representative Masson’s trichrome-stained hearts at 14 moths of age are shown. B) Masson’s trichrome-stained 6-month-old hearts show collagen in blue (left). The percentage of blue-stained area (% Blue) is significantly increased in *Tmem135* TG hearts compared to that in WT hearts (n = 4 WT, n = 4 TG; p<0.05 by t-test) (center). Immunohistochemistry of 6-month-old heart tissue indicates increase in the collagen III protein (right). C) WGA staining (green, far left) of transverse sections was used to quantify cardiomyocyte size (outlined by WGA staining) and extra-cellular space. While the cardiomyocyte size was comparable between *Tmem135* TG and WT mice (n = 6 WT, n = 6 TG, >200 cells per sample examined, p = 0.0642 by t-test), extracellular space is significantly increased in *Tmem135* TG hearts (n = 6 WT, n = 6 TG; p<0.05 by t-test). D) Survival curves show that *Tmem135* TG mice die earlier compared to WT littermates (n = 11 WT, n = 21 TG, P < 0.0001 by Log-rank test and Gehan-Breslow-Wilcoxon test).

**Fig 2:**
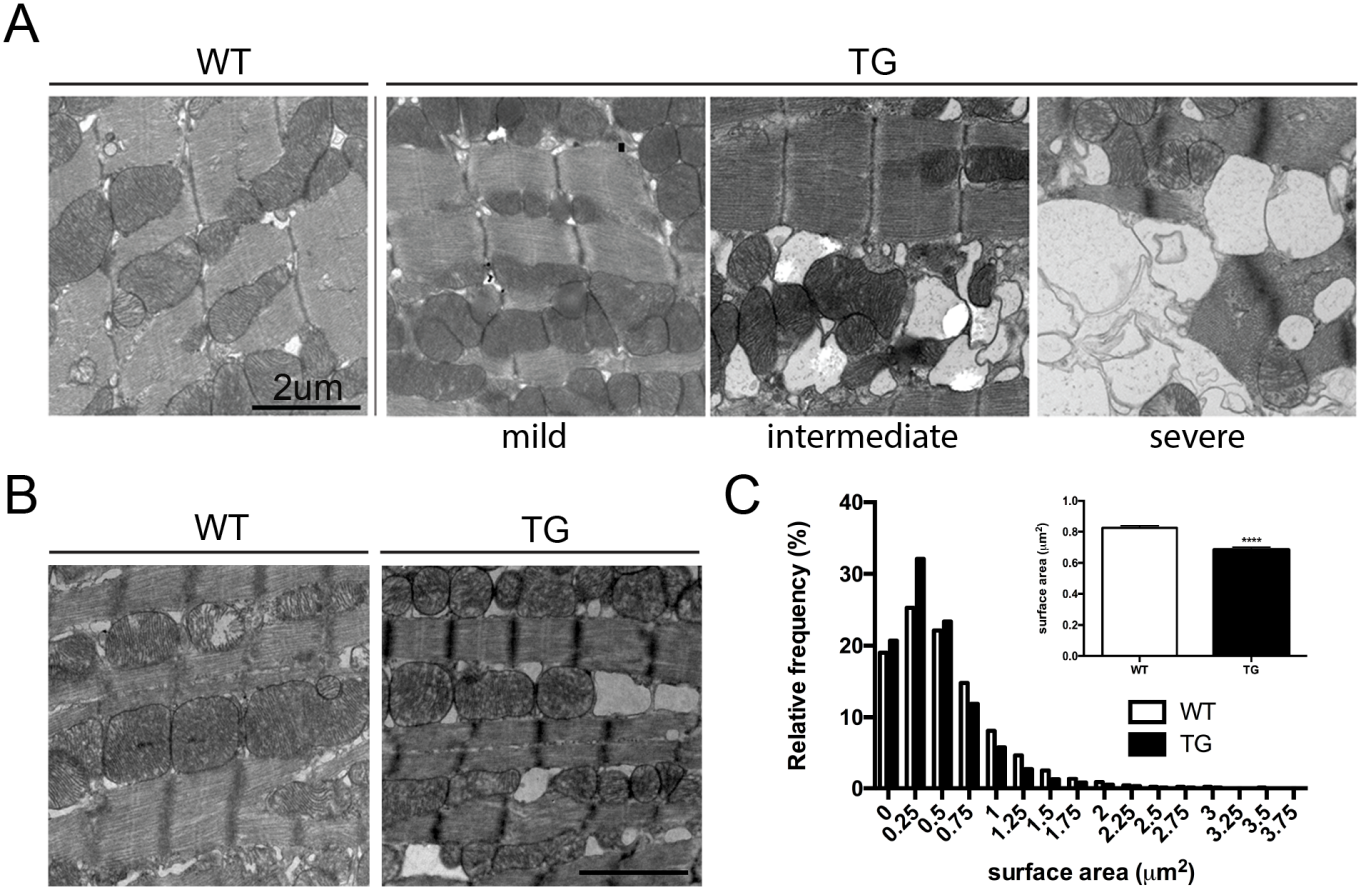
Cardiomyocyte and mitochondrial morphology are affected in *Tmem135* TG hearts. A) Electron micrographs of WT and *Tmem135* TG heart tissues at 7 months of age show varying severity of vacuolization in the cardiomyocytes of *Tmem135* TG mice (7100X). B) Electron micrographs indicate decreased mitochondrial size in *Tmem135* TG cardiomyocytes (7100X). C) Quantification of the cardiomyocyte size (n = 4 WT [8655 mitochondria examined], n = 4 TG [8950 mitochondria examined]) shows significant decrease in the average size (inset, p<0.01 by t-test), and size distribution of mitochondria in *Tmem135* TG hearts (p<0.0001 by Kolmogorov-Smirnov test).

In order to assess the effect of *Tmem135* overexpression on the heart, we compared the heart from adult transgenic mice over-expressing wild-type *Tmem135* (*Tmem135* TG) under the control of the ß-actin promoter [11] to that of age-matched, littermate non-transgenic control (WT) mice. Comparison of the mean heart weight to body weight ratio (n = 7 WT, n = 9 TG) at 14 months of age indicated 27% increase in the heart size relative to the body size in *Tmem135* TG adult mice compared to WT mice although the difference did not reach statistical significance by unpaired t-test with Welch’s correction (p = 0.085) (Fig 1A). We observed significantly greater variance in the heart weight to body weight ratio among the *Tmem135* TG group compared to the WT group (p<0.01 by F-test), possibly reflecting the variability in the genetic background of mice which is the mixture of FVB/NJ and C57BL/6J. Gross histological examination did not show marked morphological changes in the *Tmem135* TG heart (Fig 1A). However, closer examination revealed pathological changes. We examined the amount of collagen accumulation by measuring the percentage of blue-stained area in the Masson’s trichrome-stained heart sections. We observed that the *Tmem135* TG heart had significantly increased collagen compared to the WT heart at 6 months of age (Fig 1B; n = 4 WT, n = 4 TG, p<0.05 by t-test) indicating increased fibrosis, which is often associated with cardiac hypertrophy [28–30]. We also conducted immunohistochemistry using a collagen III antibody, which is a marker for extracellular collagen and found that it was also increased in the *Tmem135* TG heart (Fig 1B). Since cardiac hypertrophy could be due to cardiomyocyte hypertrophy, we examined the size of cardiomyocytes outlined by WGA staining in transverse sections of the heart at 14 months of age (Fig 1C). The analysis did not indicate a change in the size of cardiomyocytes in the *Tmem135* TG heart compared to the WT heart (Fig 1C; n = 6 WT, n = 6 TG, p = 0.0642 by t-test). These results suggest that mild cardiac hypertrophy in *Tmem135* TG mice is mainly due to increased fibrosis/collagen accumulation. We, then, conducted electron microscopy to assess changes in the ultrastructure of individual cardiomyocytes. Electron micrographs of the left ventricle muscle tissue showed large vacuoles co-occupying the space between myofibrils with mitochondria at varying severity (Fig 2A). We also observed that when present, the vacuoles often disrupted the contacts between neighboring mitochondria and increased the space between myofibrils (Fig 2A, intermediate and severe). Variability in the severity of this phenotype may be also due to the mixed genetic background of these mice. We examined the survival of *Tmem135* TG mice and found that they died earlier than WT controls (Log-rank test, P < 0.0001; Gehan-Breslow-Wilcoxon test, P < 0.0001) beginning around 4 months, and the majority of *Tmem135* TG mice succumb before 1.5 years (Fig 1D). The heart abnormalities may contribute to the earlier deaths observed in *Tmem135* TG mice.

A previous study showed that TMEM135 is a novel regulator of mitochondrial dynamics [11]. Specifically, *in vitro* overexpression of *Tmem135* resulted in a mitochondrial dynamic shift towards increased fission, which resulted in smaller mitochondria in *Tmem135* TG fibroblast cells [11]. Therefore, we tested whether mitochondrial morphology is affected in the myocardium of *Tmem135* TG mice (Fig 2B). We measured the surface area of mitochondria in electron micrographs of cardiomyocytes in 7-month-old *Tmem135* TG (n = 4, 8950 mitochondria examined) and WT (n = 4, 8655 mitochondria examined) hearts (Fig 2C). The average mitochondria size was decreased in the *Tmem135* TG heart compared to the WT heart (Fig 2C, inset, p<0.01 by t-test). Additionally, the size distribution of the mitochondria was shifted towards smaller sizes in the Tmem135 heart (Fig 2C, p<0.0001 by Kolmogorov-Smirnov test). These data suggest that mitochondrial dynamics are shifted towards increased fission in the *Tmem135* TG cardiomyocytes *in vivo*.

**Fig 3:**
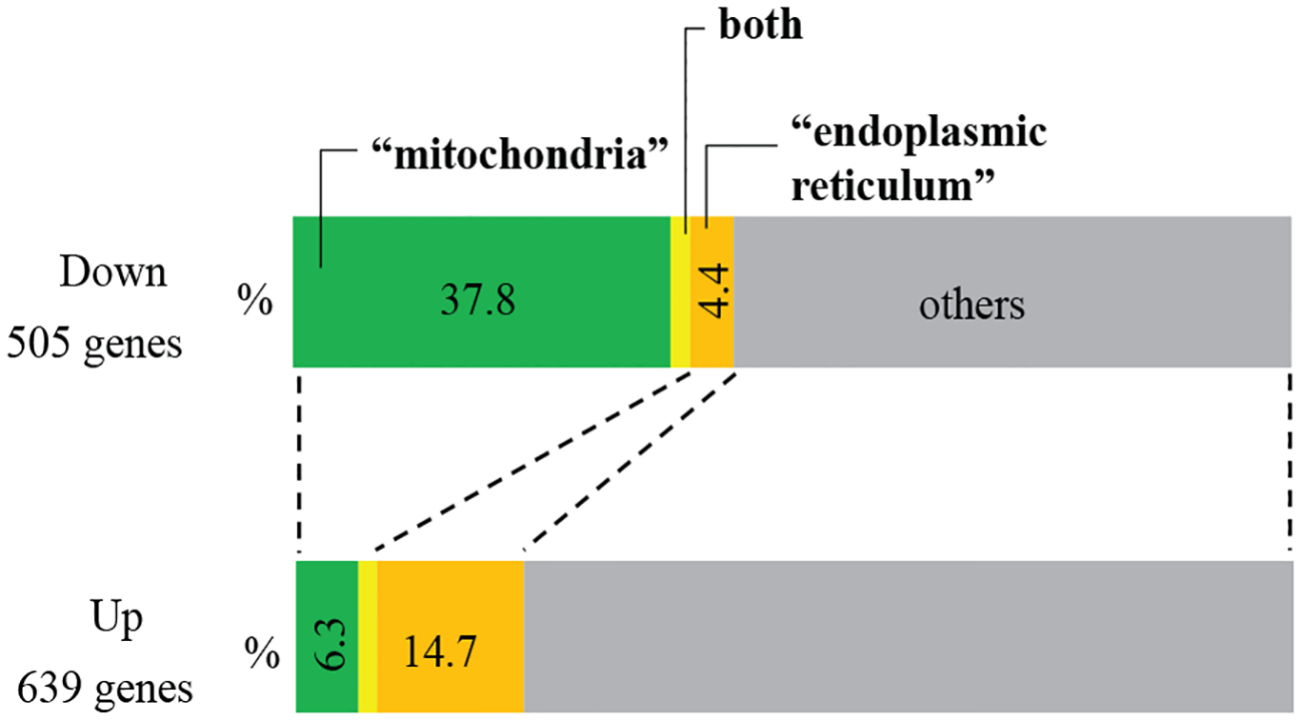
RNA sequencing shows significantly affected pathways in the *Tmem135* TG heart. RNAseq was performed on the heart of 6-month old WT and *Tmem135* TG mice (n = 3 WT, n = 3 TG). Compared to the WT heart, 505 genes were down-regulated and 639 genes were up-regulated in the *Tmem135* TG heart. Down-regulated genes were enriched for mitochondria-related functions while up-regulated genes were enriched for genes related with endoplasmic reticulum.

To identify molecular pathways affected in the *Tmem135 TG* heart, we conducted RNA sequencing (RNAseq) of 6-month-old *Tmem135* TG and WT hearts to compare gene expression profiles. We detected 1,144 differentially expressed (DE) genes between TG and WT, where 505 genes were decreased, and 639 genes were increased in the *Tmem135* TG heart compared with WT controls (Fig 3). Using the Database for Annotation, Visualization, and Integrated Discovery Functional Annotation Tool (DAVID) for gene ontology (GO) term analysis, we categorized DE genes in the *Tmem135* TG heart (Table 1). We observed over-representation of the mitochondria-associated GO terms in the down-regulated gene set. Down-regulation of mitochondria-associated genes is consistent with mitochondrial dysfunction [11] along with fragmentation observed *in vitro* in *Tmem135* TG fibroblasts [11] as well as *in vivo* in the *Tmem135* TG heart (Fig 2B and 2C). GO terms associated with the endoplasmic reticulum (ER) and stress response as well as those associated with the extracellular matrix are enriched in the up-regulated genes for *Tmem135* TG hearts (Table 2).

**Table 1:** Top 10 GO terms enriched in the down-regulated gene list of *Tmem135* TG mice.

GO root category	rank	GO term	G enes (n)	EASE score
Biological Process	1	**generation of precursor metabolites and energy**	68	3.36E-48
	2	**electron transport chain**	45	7.62E-41
	3	**oxidation reduction**	83	2.45E-33
	4	**cellular respiration**	18	7.83E-14
	5	**energy derivation by oxidation of organic compounds**	21	6.21E-13
	6	establishment of localization	112	5.08E-11
	7	transport	111	7.46E-11
	8	**oxidative phosphyrylation**	15	1.06E-10
	9	coenzyme metabolic process	22	1.20E-10
	10	metabolic process	242	2.91E-10
Cellular Component	1	**mitochondrion**	189	3.30E-93
	2	**mitochondrial part**	119	3.00E-78
	3	organelle innermembrane	90	6.87E-68
	4	**mitochondrial inner membrane**	88	1.46E-67
	5	**mitochondrial membrane**	92	2.26E-63
	6	**mitochondrial envelope**	93	5.31E-62
	7	organelle envelope	98	5.73E-54
	8	envelope	98	8.15E-54
	9	cytoplasmic part	248	2.50E-46
	10	**respiratory chain**	38	7.57E-42
Molecular Function	1	**oxidoreductase activity**	72	4.71E-23
	2	catalyic activity	203	2.50E-13
	3	monovalent inorganic cation transmembrane transporter activity	20	5.94E-13
	4	**hydrogen ion transmembrane transporter activity**	19	2.22E-12
	5	inoraganic cation transmembrane activity	21	8.39E-11
	6	conezyme binding	23	1.45E-10
	7	**NADH dehydrogenase (ubiqinone) activity**	11	1.64E-10
	8	**NADH dehydrogenase activity**	11	1.64E-10
	9	**NADH dehydrogenase (quinone) activity**	11	1.64E-10
	10	cofactor binding	27	1.74E-10

Down-regulated GO terms ranked by biological process, cellular component, and molecular function. Bold terms are those associated with mitochondrial energy production.

**Table 2:** Top 10 GO terms enriched in the upregulated gene list of *Tmem135* TG mice.

GO root category	rank	GO term	G enes (n)	EASE score
Biological Process	1	cellular adhesion	51	8.49E-10
	2	biological adhesion	51	9.09E-10
	3	oxoacid metabolic process	44	1.14E-08
	4	carboxylic acid metabolic process	44	1.14E-08
	5	organic acid metabolic process	44	1.20E-08
	6	inflammatory response	28	1.95E-08
	7	response to stress	79	2.24E-08
	8	cellular ketone metabolic process	44	2.35E-08
	9	response to unfolded protein	14	2.76E-08
	10	response to wounding	35	5.05E-08
Cellular Component	1	extracellular region	150	3.71E-29
	2	extracellular region part	91	3.23E-25
	3	extracellular matrix	57	3.60E-25
	4	proteinaceous extracellular matrix	54	1.54E-23
	5	extracellular matrix part	24	4.03E-14
	6	endoplasmic reticulum	74	1.44E-13
	7	endoplasmic reticulum part	29	6.66E-09
	8	cytoplasmic part	200	6.89E-08
	9	collagen	9	9.88E-08
	10	basement membrane	15	1.52E-07
Molecular Function	1	glycosaminoglycan binding	24	2.78E-12
	2	pattern binding	25	4.90E-12
	3	polysaccharide binding	25	4.90E-12
	4	heparin binding	20	2.15E-11
	5	carbohydrate binding	37	1.22E-10
	6	protein binding	259	5.40E-09
	7	growth factor binding	16	1.13E-08
	8	binding	430	1.84E-07
	9	extracellular matrix structural constituent	10	3.60E-07
	10	platelet-derived growth factor binding	6	4.53E-06

Upregulated GO terms ranked by biological process, cellular component, and molecular function

**Fig 4:**
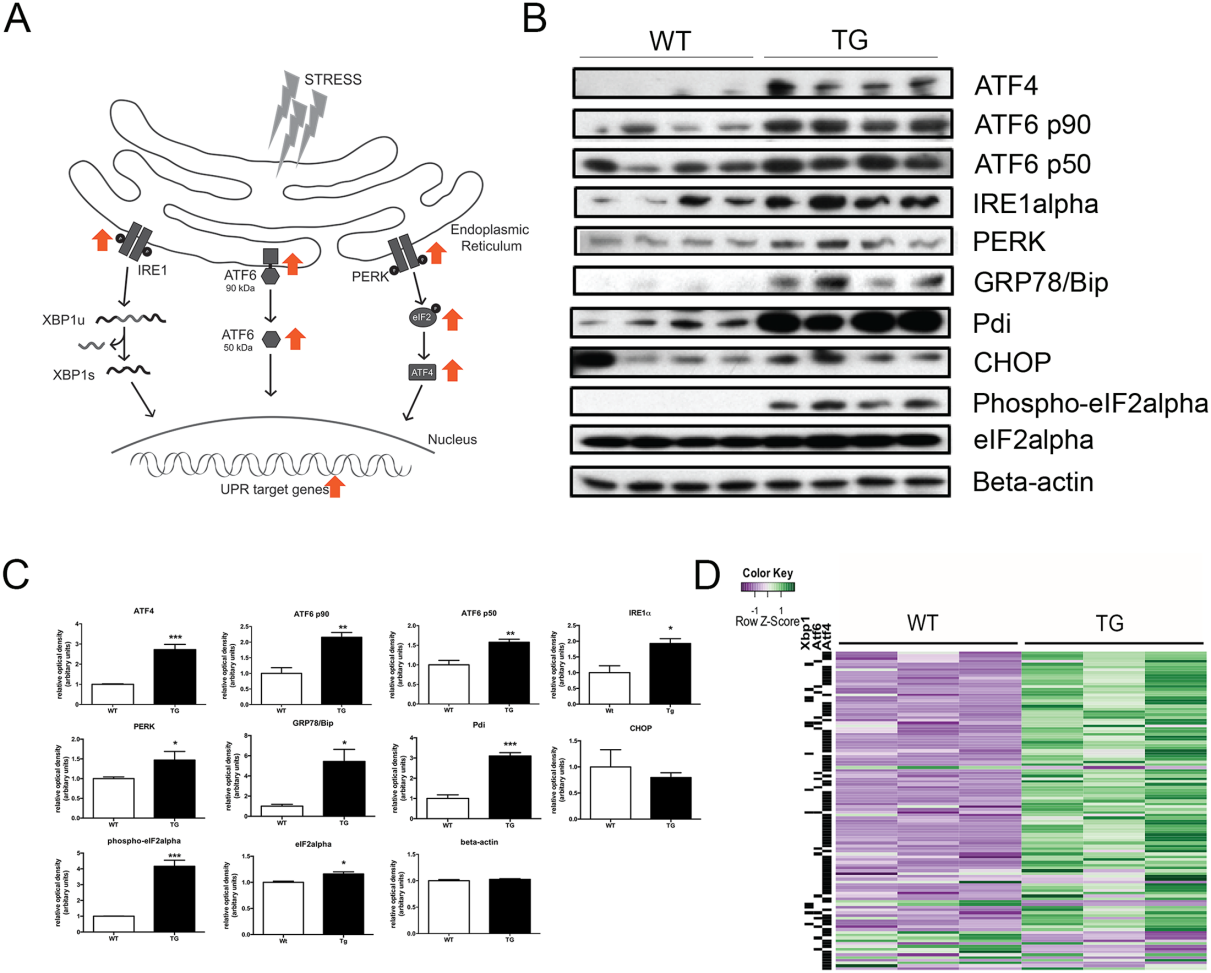
ER stress pathways are affected in *Tmem135* TG hearts. A) Schematic of the unfolded protein response (UPR) activated by ER stress. Orange arrows indicate upregulation in *Tmem135* TG hearts. B) Western blot analysis of UPR proteins in WT and *Tmem135* TG hearts at 2 months of age. C) Quantification of UPR proteins shows significant increase in most of these proteins in *Tmem135* TG hearts compared to WT hearts (n = 4 TG, n = 4 WT). *p<0.05, **p<0.01, ***p<0.001 by t-test. D) Heat map of 127 downstream targets of ATF4, Xpb1, and ATF6, the major transcription factor from each branch of the UPR. Note that a majority of these genes (88 out of 127) are ATF4 targets.

The GO term analysis suggested that the ER stress may play a role in the development of *Tmem135* TG heart phenotypes. Genes in all three branches of the unfolded protein response (UPR) activated by ER stress [31] were increased in *Tmem135* TG hearts (Fig 4A). We conducted western blot analysis to test the protein expression of key ER stress markers including protein kinase RNA-like endoplasmic reticulum kinase (PERK) [32], phosphorylated PERK, inositol-requiring protein-1 (IRE1α) [33], 78-kilodalton Glucose Regulated Protein (GRP78)/ binding immunoglobulin protein (Bip) [34], protein disulfide isomerase (Pdi) [35,36], eukaryotic translation initiation factor (eIF2α) [37], and phosphorylated eIF2α as well as key UPR transcription factors activating transcription factor (ATF)6 [38], and ATF4 [39]. We performed this analysis at 2 months of age, in order to also test whether increase in the ER stress response is observed at an earlier timepoint. We found that a majority of these ER stress markers are increased in the *Tmem135* TG heart compared to the WT heart at 2 months of age (Fig 4B and 4C, S1 Fig). These results indicate that the UPR is activated by increased ER stress in the *Tmem135* TG mouse heart at an early stage of phenotype progression, further suggesting its involvement in the development of the heart phenotypes. In addition, we examined whether expression of target genes downstream of primary UPR is affected in the *Tmem135* TG heart using RNAseq data. The analysis identified known targets of UPR transcription factors in the DE gene set, most of which are upregulated in the *Tmem135* TG heart (Fig 4A and 4D). A majority of these target genes (88 genes) are direct targets of ATF4 (Fig 4D), suggesting that ATF4 is the dominant UPR transcription factor activated by ER stress in the *Tmem135* TG heart.

**Fig 5:**
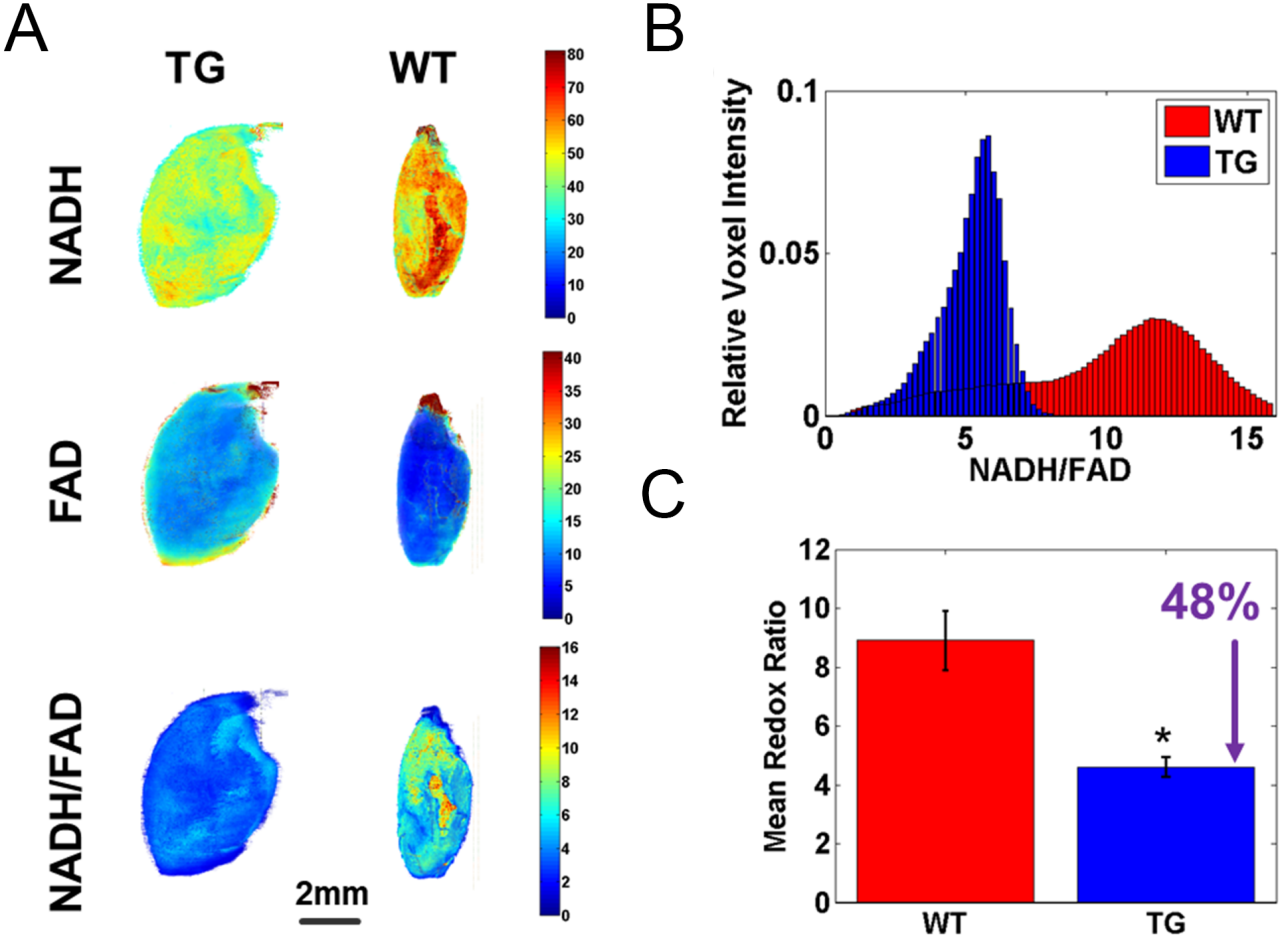
The redox state of *Tmem135* TG hearts is affected. A) Representative 3D rendered cryo-images of hearts from *Tmem135* TG and WT mice. The fluorescence patterns for reduced nicotinamide adenine dinucleotide (NADH), flavin adenine dinucleotide (FAD), and the tissue redox ratio (NADH/FAD) are shown. B) Corresponding histograms of voxel distribution of the redox ratio images. C) Bar plot of mean ± STD for volumetric histograms showing statistical difference (n = 7 WT, n = 7 TG, p<0.001 by t-test).

Oxidative stress is considered as one of the factors that could trigger ER stress [40]. Our previous study showed increased reactive oxygen species (ROS) in cultured *Tmem135* TG fibroblasts [11], which may be also increased in the *Tmem135* TG heart. Using optical cryo-imaging [23–25], we examined the redox state of the *Tmem135* TG heart (Fig 5). This method detects changes in the oxidation state of mitochondrial metabolic coenzymes NADH (NAD in the reduced form) and FAD (FADH_2_ in its oxidized form), and provides a quantitative marker for oxidative stress (the ratio of NADH/FAD; the redox ratio) in tissues [23–25]. The 3D cryo-imaging revealed lower NADH and higher FAD fluorescence signals in *Tmem135* TG hearts compared with WT hearts (Fig 5A) resulting in lower redox ratio (Fig 5B and 5C) (n = 7 WT, n = 7 TG, p<0.001 by t-test). These results indicate higher levels of oxidative stress in *Tmem135* TG hearts compared with WT hearts, and suggest the possibility that increased oxidative stress may trigger ER stress in the *Tmem135* TG heart.

**Fig 6:**
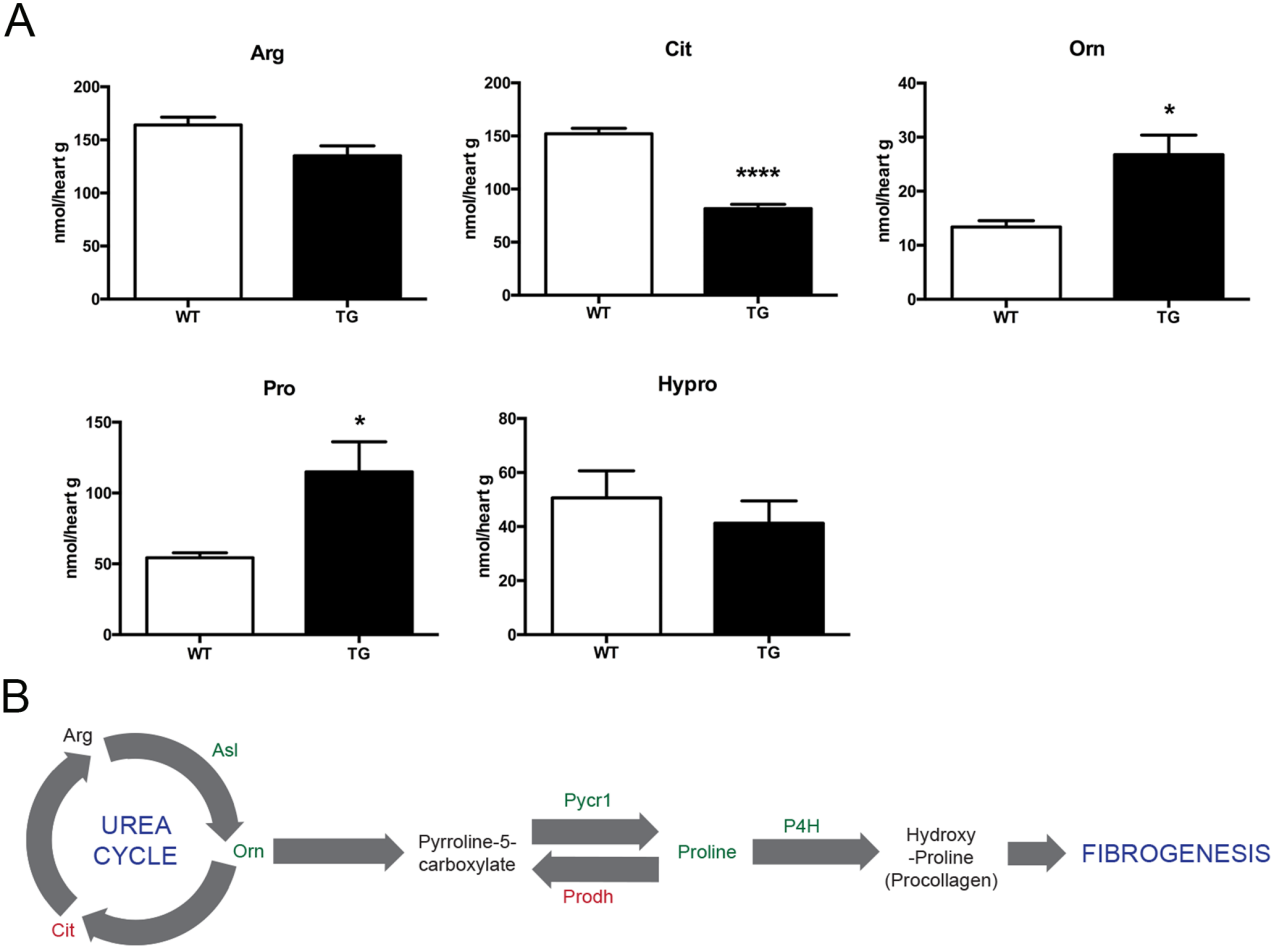
Collagen synthesis pathways are affected in *Tmem135* TG mice. A) Quantification of small molecules in the heart lysate of 2-month-old WT and *Tmem135* TG mice shows significant increase of molecules in the collagen synthesis pathway (Orn and Pro) in the *Tmem135* TG heart (n = 4 WT, n = 4 TG; *p<0.05, ****p<0.0001 by t-test). Orn: ornithine, Pro: proline, Cit: citrulline, Hypro: hydroxyproline. B) Schematic of the collagen synthesis pathway leading to fibrogenesis depicting significantly affected molecules and enzymes in *Tmem135* TG hearts. (green = increased, red = decreased, black = unchanged).

The GO term analysis of the RNAseq data also showed a significant enrichment of genes associated with the extracellular matrix (Table 2) in the *Tmem135* TG heart, which is consistent with the histological data showing increased collagen (Fig 1C). In order to examine the collagen synthesis pathway, we conducted qPCR analysis on genes associated with the procollagen pathway. The RNA sequencing results showed that mRNA for enzymes involved in the procollagen production (arginosuccinate lyase [ASL], pyrroline-5-carboxylate reductase 1 [PYCR1], and prolyl 4-hydroxylase [P4H]) were up-regulated, while an enzyme that suppresses collagen production, proline dehydrogenase (PRODH), was down-regulated in the *Tmem135* TG heart. Furthermore, some of the secondary metabolites within the urea cycle pathway and the procollagen production pathway, ornithine (Orn) and proline (Pro), were increased in the *Tmem135* TG heart (Fig 6A). These data indicate that the molecular pathway leading to collagen accumulation is activated in the *Tmem135* TG heart (Fig 6B).

**Fig 7:**
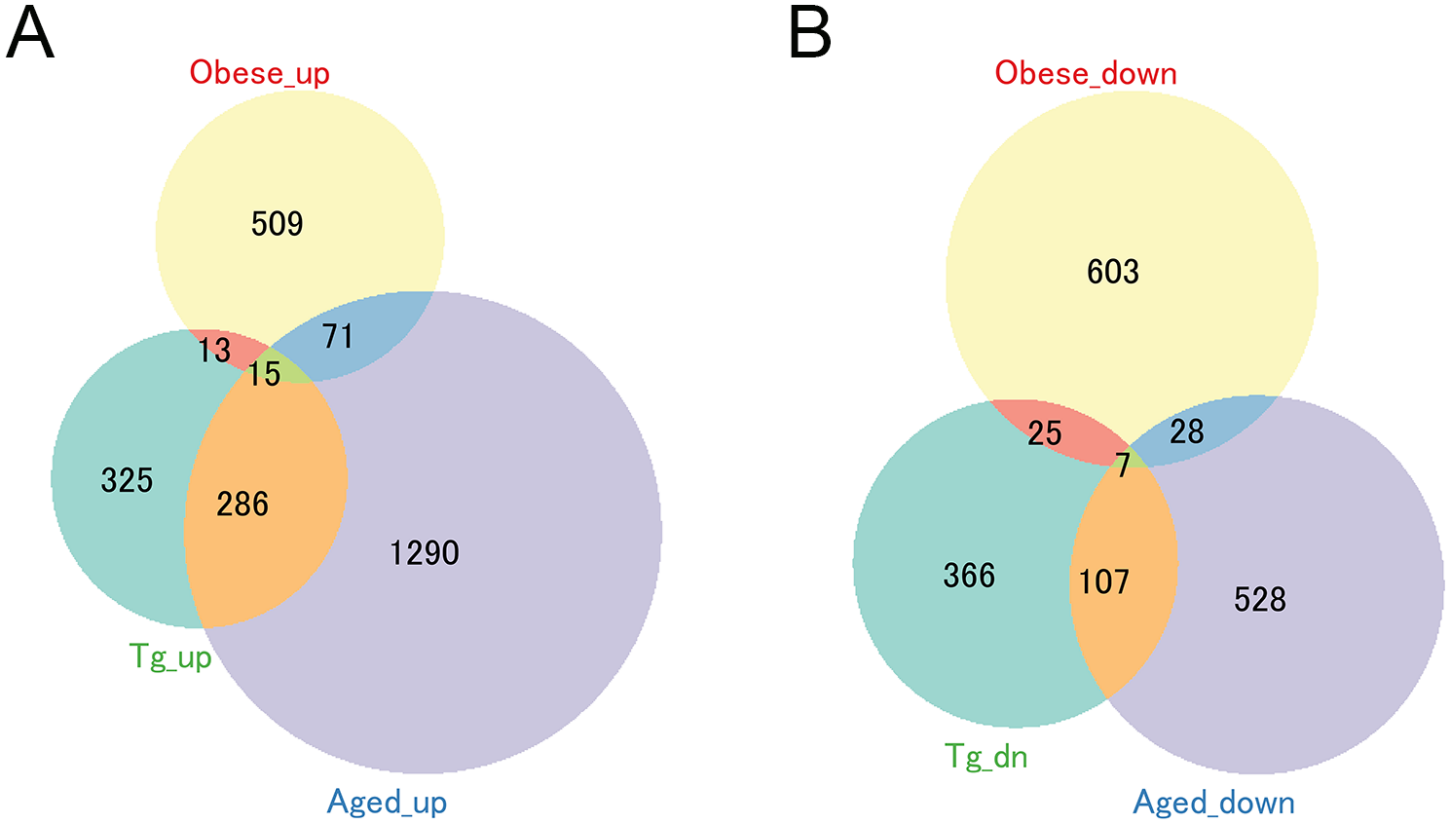
The profile of DE genes in *Tmem135* TG hearts is similar to that of aged hearts. Comparison of DE gene sets of hearts from *Tmem135* TG mice, aged (23–25 month-old) mice, and a diet-induced obesity mouse model. *GeneOverlap* simulation in R confirmed significant overlap in the up-regulated (A) and down-regulated (B) DE gene sets of *Tmem135* TG and aged mouse hearts. p = 7.6e-167 and p = 1.3e-65, respectively, by Fisher’s Test.

Pathologies in the *Tmem135* TG heart including hypertrophic cardiomyopathy and collagen accumulation (Fig 1A and 1B) indicative of fibrosis are also observed in the aging heart [41]. Moreover, TMEM135 was found to be involved in the regulation of the aging process in the retina [11]. Therefore, we compared the gene expression profile of the *Tmem135* TG heart with publicly available datasets for the aged heart. We obtained gene expression data of the heart of 25–28 month old C57BL/6J WT mouse heart (GSE12480) and those of a diet-induced obesity model (GSE47022) [21] as well as those from the respective control mice (young [4–6 month old] C57BL/6J WT mice and non-obese mice on control diet, respectively) from public database. We determined the DE gene sets of each pathology model compared to controls, and then determined the similarity of the DE genes between the *Tmem135* TG heart and the other 2 models using *GeneOverlap* in R programming language. Through this analysis, we found that DE genes in the 6-month old *Tmem135* TG mouse heart significantly overlaped with DE genes in aged (25–28 months old) WT mouse heart. Among up-regulated DE genes, 301 genes were found to overlap between the *Tmem135* TG heart and aged heart while 86 genes overlap between the obese model and the aged heart and 28 genes overlap between the *Tmem135* TG heart and the obese model (Fig 7A). Similarly, among down-regulated DE genes in each condition, 114 overlapped genes were found between the *Tmem135* TG and aged heart while 35 genes overlapped between the obese model and the aged heart and 32 genes overlapped between the *Tmem135* TG heart and the obese model (Fig 7B). Using *GeneOverlap* simulation in the R package, we confirmed significant overlap between DE gene sets of *Tmem135* TG heart and the aged heart (p = 7.6e-167 and p = 1.3e-65 for upregulated and downregulated genes, respectively). In addition, we performed function-based comparison of DE genes in the *Tmem135* TG and aged heart using over-represented gene ontology (GO) terms. Using the DAVID analysis, we found that 65% and 29% of GO terms enriched in up-regulated and down-regulated DE (respectively) in the *Tmem135* TG heart overlap with those enriched in the aged heart. This further suggested functional correlation between the *Tmem135* TG mouse heart and aged heart. Thus, the *Tmem135* TG mouse heart shares common features with the aged heart not only in pathological phenotypes but also in the gene expression profiles.

## Discussion

In this study, we investigated the effect of overexpression of the *Tmem135* gene, which encodes a novel regulator of mitochondrial dynamics [11], in the heart. Overexpression of *Tmem135* affects the size of mitochondria in cardiomyocytes *in vivo* and leads to pathological phenotypes in the heart including collagen accumulation and hypertrophy. Furthermore, the gene expression analysis showed that genes associated with the ER stress pathway are upregulated in the *Tmem135* TG heart. It also showed that gene expression changes in the heart of *Tmem135* TG mice significantly overlap with those of aged mice, suggesting that mitochondrial dynamics may be involved in the normal aging process of the heart. Thus, our study revealed the pathological consequence of dysregulated mitochondrial dynamics due to overexpression of *Tmem135*, and suggested downstream molecular changes that may underlie those disease pathologies.

### TMEM135 as a mitochondrial fission factor in the heart

TMEM135 was recently identified as a novel factor that regulates mitochondrial dynamics [11]. Overexpression of *Tmem135* induces increased fragmentation of mitochondria in fibroblast cells (*in vitro*) [11] and in the retinal tissue (*in vivo*) (Ikeda A: unpublished data). Our study in the heart showed that the mitochondrial size in cardiomyocytes of *Tmem135* TG mice is decreased compared to WT mice (Fig 2B and 2C), indicating that TMEM135 has the same function in cardiomyocytes to induce mitochondrial fission. Thus, *Tmem135* TG mice provide a new model to study the role of mitochondrial dynamics in the heart. The consequence of dysregulated mitochondrial dynamics in the heart has been investigated by targeting genes involved in mitochondrial dynamics in mice. Using *Drp1* and *Mfn1*/*Mfn2* conditional KO mice, Song et al. showed that mitochondrial dynamics defects lead to heart disease phenotypes [10]. When *Drp1* is ablated in the adult mouse heart causing hyper-fused mitochondria, the mice show dilated cardiomyopathy, while ablation of both *Mfn1* and *Mfn2* in the adult mouse heart causes a decrease in mitochondrial fusion leading to hypertrophy of the heart [10]. These results suggest the importance of balanced mitochondrial dynamics in maintaining normal structures and functions of the heart tissue. In another study, imbalanced processing of mitochondrial dynamin-like GTPase OPA1 causes fragmentation of mitochondria and leads to dilated cardiomyopathy [42]. Although cardiac *Mfn1*/*Mfn2* deficiency, induced processing of OPA1, and *Tmem135* overexpression all result in fragmentation of mitochondria in cardiomyocytes, the phenotypes of the heart in these models are distinct. *Mfn1*/*Mfn2* deficient mice [10] and *Tmem135* TG mice (Fig 1A) show cardiac hypertrophy while mice with induced processing of OPA1 develop dilated cardiomyopathy [42]. Collagen accumulation indicative of cardiac fibrosis is observed in *Tmem135* TG mice (Fig 1B) and mice with induced OPA1 processing [42], but not in *Mfn1*/*Mfn2* deficient mice [10]. Most recently, it was also reported that mitochondrial fragmentation due to cardiac overexpression of *Drp1* did not result in any cardiac pathologies [43]. These differences could be possibly due to multiple molecular pathways affecting mitochondrial fusion and fission in the heart, and thus producing different pathological consequences depending on which pathway is affected to cause fragmentation of mitochondria. Additionally, the genetic background of mice could be another factor that potentially affects phenotypic differences considering that these mouse models were on different genetic backgrounds. *Mfn1*/*Mfn2* deficient mice [10] were on a C57BL/6J background, while mice with induced OPA1 processing [42] had a mixture of C57BL/6 and FVB/N backgrounds. Mice with cardiac overexpression of *Drp1* [43] were generated by crossing *Drp1* transgenic mice on a FVB/N background with *myh6* promoter-driven doxycycline-suppressible line whose genetic background was not described [44]. *Tmem135* TG mice used in the current study were on a mixed background of FVB/NJ and C57BL/6J. We observed variability in the severity of phenotypes among these mice (Figs 1A and 2A), further suggesting the contribution of genetic factors on the severity of cardiac abnormalities caused by defective mitochondrial dynamics. Investigation into the downstream molecular changes as well as genetic factors that affect phenotype manifestations in these mouse models could further reveal how mitochondrial dynamics affect the structure and function of the heart.

### ER stress is upregulated in the *Tmem135* TG heart

The GO Term analysis of RNAseq data indicated that ER stress-related genes constitute a large proportion of upregulated genes in the *Tmem135* TG heart. In addition, we observed upregulation of ER stress associated factors at the protein level (Fig 4B and 4C). These results indicate that ER stress is upregulated in the *Tmem135* TG heart. The relation between mitochondrial dynamics and ER stress has been reported in different tissues. Disruption of *Mfn2* has been shown to increase ER stress in fibroblasts and cultured cardiomyocytes [45] while inhibition of DRP1 alleviates stress in cells [46]. Filippi et. al also showed that increased mitochondrial fission by constitutively activating DRP1 is sufficient to induce ER stress in the brain [47]. These findings suggest that an imbalance toward mitochondrial fission induces ER stress. In case of the *Tmem135* TG heart, it may be also true that mitochondrial fragmentation induced by overexpression of *Tmem135* initiates the ER stress response. It is possible that increased oxidative stress due to dysfunctional mitochondria mediate this process. In addition to our earlier observation of dysfunctional mitochondria and increased ROS in cultured *Tmem135* TG fibroblasts [11], our optical cryo-imaging indicated significantly increased oxidative stress in *Tmem135* TG hearts (Fig 5). Since there is accumulating evidence that ER stress signaling is elicited in response to oxidative triggers [40], our results suggest increased oxidative stress due to mitochondrial abnormalities may trigger ER stress in the *Tmem135* heart. On the other hand, a number of studies indicated communication between ER and mitochondria [48]. ER stress has been shown to increase mitochondrial ROS generation [40,49,50], and can cause functional defects in mitochondria as observed in diseases such as neurodegeneration [51] and multiple sclerosis [52]. Therefore, it is possible that mitochondrial defects and ER stress enhance each other to make the phenotypes more severe in this fashion. The *Tmem135* TG mouse model can be used to investigate the molecular pathways affected by increased mitochondrial fission and ER stress, and how they may interact with each other to cause heart pathologies *in vivo*.

### The ATF4 pathway is specifically activated in the *Tmem135* TG heart

In *Tmem135* TG mice, the expression of genes and proteins involved in three major UPR pathways are up-regulated. Among those pathways, the ATF4 pathway especially shows major changes. First, we observed very little expression of the ATF4 protein and its activator, phosphorylated eIF2**α** [53] in the WT heart, while their expression is largely upregulated in the *Tmem135* TG heart (Fig 4B and 4C). Secondly, known ATF4 targets also show the most significant changes among targets of the UPR pathways (Fig 4D). Thus, in the *Tmem135* TG model, ATF4 may play a major role in mediating ER stress in the heart. Recent studies have linked mitochondrial abnormalities to induction of ER stress [48] and activation of ATF4 [54,55]. In addition to ER stress, ECM-related genes are highly represented in the DE gene set of the *Tmem135* TG heart. This is consistent with the histological data showing increased collagen in the *Tmem135* TG heart (Fig 1B), indicative of ECM remodeling. Upon further investigation of the procollagen pathway, we observed increases in metabolites and expression of enzymes that facilitate the production of procollagen (Fig 6). ER stress and activation of the UPR have been suggested as a pro-fibrotic stimulus in the heart as well as other internal organs [56]. Increased ATF4 activity in the *Tmem135* TG heart may possibly be the cause of fibrosis in the *Tmem135* TG heart, since pyrroline-5-carboxylate reductase 1 (PYCR1), which is upregulated in the *Tmem135* TG heart, is a key enzyme for synthesis of collagen, and its expression has been shown to depend on ATF4 [57]. Further investigation using conditional knockout mice for *Atf4* combined with *Tmem135* TG mice may reveal the role of ATF4 in pathogenesis in the *Tmem135* TG heart.

### The *Tmem135* TG heart shows similar gene expression profiles and pathologies to aging hearts

Alterations in the balance of mitochondrial dynamics have been linked to aging and age-dependent disease phenotypes. For instance, in mouse skeletal muscle, elongation of the mitochondria has been linked to aging-associated phenotypes [58]. In the kidney of diabetic mice, mitochondrial fragmentation precedes the development of histological damages observed in kidneys [59]. Both cancer and neurodegenerative disease are linked to dysregulated mitochondrial dynamics [60,61]. In this study, we found that the *Tmem135* TG heart displays some of the aging and age-related disease phenotypes in the heart including hypertrophy and collagen accumulation indicative of fibrosis (Fig 1A and 1B). In addition, we compared the gene expression profile of the *Tmem135* TG heart to that of the aged mouse heart, which showed a significant overlap of genes between *Tmem135* TG and aged hearts (Fig 7). Consistent with the phenotypic overlap in collagen accumulation/fibrosis, ECM-related genes were found in the overlap. Mitochondria-related genes were also prominent within the overlap, suggesting that changes that occur in mitochondrial functions during the process of aging may be involved in the development of aging-associated heart pathologies. Thus, *Tmem135* TG may provide a mouse model in which certain aspects of heart aging is accelerated.

## Supporting information

S1 Fig Phosphorylated PERK in *Tmem135* TG hearts. A) Western blot analysis for phosphorylated PERK in WT and *Tmem135* TG hearts at 2 months of age. B) Quantification of phosphorylated PERK shows a trend of increase in *Tmem135* TG hearts compared to WT hearts although it did not reach statistical significance (n = 5 TG, n = 5 WT, p = 0.056 by t-test).
(TIF)
