# Supplementary figures and images for: The effect of Tmem135 overexpression on the mouse heart
Source: PLoS One. 2018 Aug 13;13(8):e0201986. doi: 10.1371/journal.pone.0201986 (PMC6089435; doi:10.1371/journal.pone.0201986)

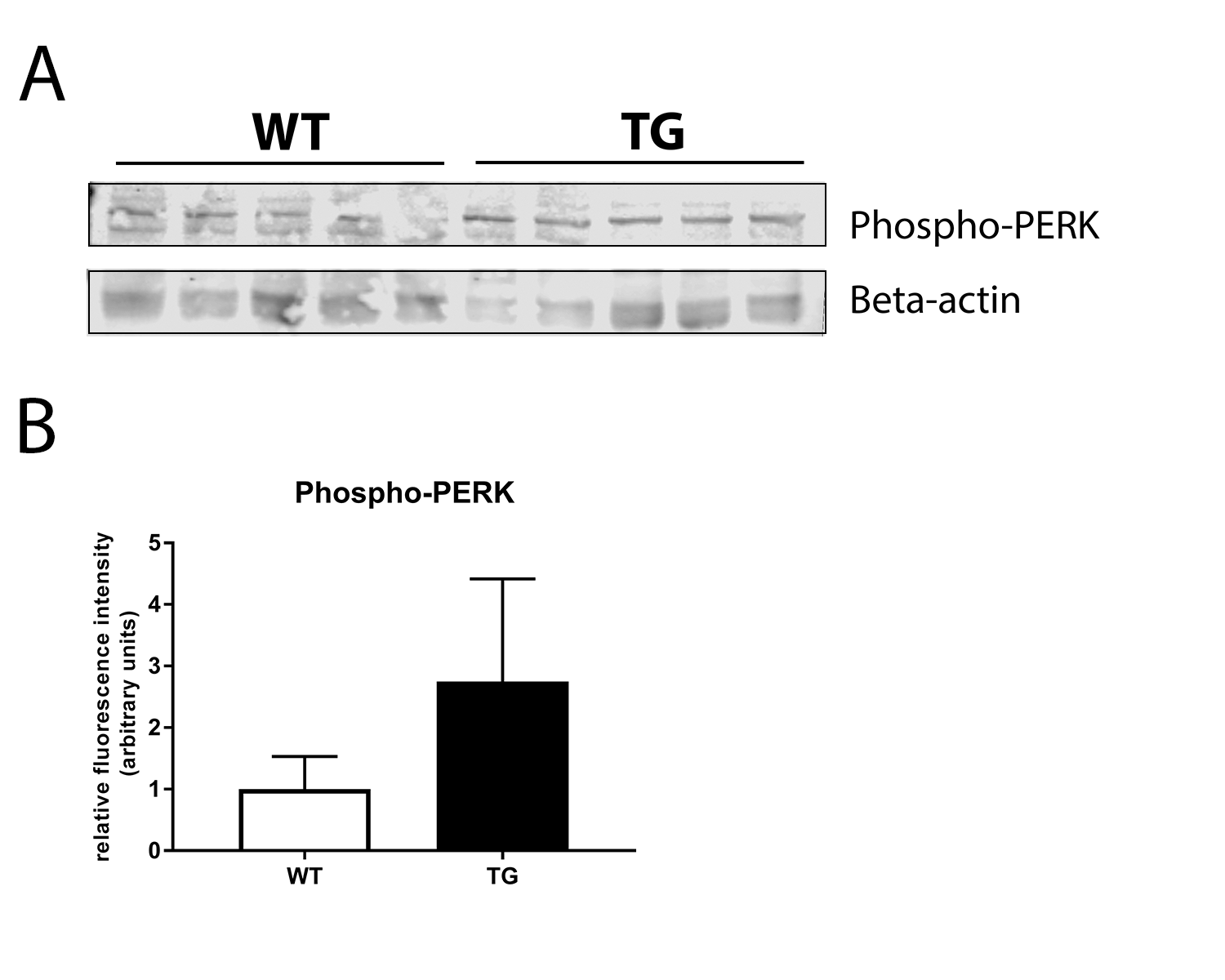

Supplement: S1 Fig — A) Western blot analysis for phosphorylated PERK in WT and Tmem135 TG hearts at 2 months of age. B) Quantification of phosphorylated PERK shows a trend of increase in Tmem135 TG hearts compared to WT hearts although it did not reach statistical significance (n = 5 TG, n = 5 WT, p = 0.056 by t-test). (TIF) [file pone.0201986.s001.tif]
